# The challenges and rewards of social prescribing in family medicine

**DOI:** 10.3389/frhs.2024.1446397

**Published:** 2024-10-14

**Authors:** Jonathan T. W. Au Eong

**Affiliations:** Lee Kong Chian School of Medicine, Nanyang Technological University, Singapore, Singapore

**Keywords:** social prescribing, family medicine, holistic care, biopsychosocial (BPS) model, social determinants of health (SDOH)

## Introduction

In the practice of medicine, it is not uncommon to observe drastically different treatment outcomes in patients with nearly identical diseases and medical treatment. This phenomenon can be attributed to a variety of factors apart from medical care that can influence health outcomes, collectively known as the social determinants of health (SDOH). These include income, education, employment status, availability of healthy food and social support (1).

The impact of SDOH on well-being and health was illustrated by McKeown et al, who studied death records in England and Wales from the mid-19th century to the early 1960s (2, 3). They observed that mortality from different causes had fallen significantly and steadily decades before the emergence of modern medical care modalities such as antibiotics and the intensive care unit. They posited that the increase in life expectancy could be attributed to improved living conditions, such as access to clean water, nutrition and sanitation (4).

It is becoming increasingly clear that the practice of medicine goes beyond just the biomedical aspect. This is encapsulated in the “biopsychosocial” approach to medicine proposed by Engel in 1977 (5). It emphasizes the social, psychological and behavioral dimensions of illness beyond the biomedical aspect and has grown in popularity since its introduction (5).

## The practice of social prescribing

Social prescribing, also known as community referral, is the practice of connecting patients and their carers with a range of local non-medical sources of support to optimize SDOH (6). The referrals are generally, but not exclusively, from professionals in primary care settings. First practiced in the United Kingdom (UK) in the 1990s, social prescribing aims to expand the options in primary care consultation by providing locally available resources which are often within the voluntary, community and social enterprise sector (7). This allows individuals to be more connected and resilient, enabling them to take ownership of their own care.

Social prescribing is in line with the World Health Organization's (WHO) Regional Action Plan on Healthy Aging in the Western Pacific, which advocates for support of the older population through collaboration between community service providers and the community health team (8). As supporting patients whose medical issues are exacerbated by non-medical problems becomes progressively more complex, social prescribing has become increasingly relevant and important, especially in the practice of family medicine. This is because family physicians typically follow-up with patients over a long period of time and often understand their patients' social background thoroughly.

The practice of social prescribing is gradually becoming more popular because the demand for non-medical care is increasing in most societies, and social prescribing is theoretically an effective way of addressing these needs (9). This article discusses some of the potential rewards and challenges in offering non-medical care in the primary care setting.

## Rewards of social prescribing

Social prescribing has a myriad of potential benefits, among which is an improvement in well-being and health. It achieves this by incorporating a variety of social, economic and environmental considerations to address an individual's needs in a holistic way and encourages individuals to take greater ownership of their health.

### Improvement in health and well-being

According to the WHO, global life expectancy has increased significantly, from 66.8 years in 2000 to 73.4 years in 2019 (10). Loneliness and depression amplify with age, stemming from a loss of familial ties, independent living and diminishing ties to cultural roots (11). There is mounting evidence that loneliness is detrimental to health and is associated with an increased risk of coronary heart disease, stroke, depression, cognitive decline and Alzheimer's disease (12). Recent evidence suggests that loneliness is not exclusive to the elderly but affects people of all ages (13, 14). It is clear that there is a great societal need for emotional and well-being services outside of medical therapy.

A wide variety of services are available through social prescribing, including community-based activities, information and advice, befriending and community transport. These were the most frequently assessed services in the Rotherham Social Prescribing Pilot Project in the UK (15). In this regard, social prescribing emerges as a feasible solution to match community resources and activities with those facing loneliness and social isolation. A mixed methods evaluation of social prescribing in the UK found that 72% of service-users felt less lonely after receiving support, with additional benefits of improved well-being, increased confidence, and greater life purpose (16).

Beyond emotional well-being, social prescribing has positive effects on physical health as well. This is especially helpful as many of those living longer have chronic medical conditions and poor health which compromise quality of life (17). For example, green social prescribing, which aims to connect people with nature-based activities to improve physical health, has become increasingly popular. An evaluation of the Opening the Doors to the Outdoors program in north Wales, a 12-week intervention focusing on either outdoor walking or climbing activities, found that participants experienced benefits to physical health in terms of fitness and strength (18). Likewise, an appraisal of the Luton Social Prescribing Program by Pescheny et al. also found an increase in energy expenditure from all levels of physical activities after enrollment in the program (19). A social prescribing pilot in Australia for individuals living with mental illness also found that participation in social prescribing programs led to significant improvements in quality of life and health status (20).

Current literature shows a strong link between emotional well-being and physical health. A meta-analysis by Lamers et al. found that improvements to emotional well-being may improve the prognosis of physical illness (21). Social prescribing can help to alleviate social isolation and anxiety, boost social interaction and improve confidence in daily activities and health (9).

### Appropriate usage of scarce medical resources and improving patient satisfaction

In a world where medical resources are finite, there is a mismatch in the supply of medical resources and growing demand of patients (22). Appropriate resource allocation remains a central tenet of the decision-making process in any healthcare system (23). Social prescribing is a potential solution to relieve the strain on the medical system, ensuring the judicious use of limited medical resources.

The medical and non-medical issues of patients are often intricately intertwined, as complex socioeconomic factors such as income, wealth and education have a profound effect on health outcomes (4, 24). It is estimated that approximately 20% of patients in the UK consult their general practitioner for primarily social issues (25), with an expectation to find solutions to their non-medical problems.

However, primary care workers may lack the appropriate expertise or capacity to address these non-medical issues by themselves (7). This mismatch of expertise and patient expectations may result in unmet social needs, leading to repeat clinic visits, perceptions of inadequate medical care and subsequently patient dissatisfaction (9). In addition, patients referred from primary care settings to tertiary care centers with persistent unresolved social issues may require prolonged hospitalization stay. This was shown by Toh et al. in a Singapore study which found that social factors such as patients living alone, the need for financial aid and caregiver stress had significant associations with prolonged length of stay in acute hospitals (26).

Social prescribing serves as an adjunctive measure in medical care to address the non-medical needs of patients. By referring patients to social prescribing link workers, primary physicians can ensure that the non-medical concerns of patients can be addressed by professionals with the appropriate expertise, while channeling limited clinical resources towards patients' medical complaints. Furthermore, supporting patients with the appropriate resources for their non-medical issues helps to enhance patient satisfaction and also fosters trust in the healthcare sector.

## Challenges of social prescribing

Despite its potential benefits, a number of challenges pose barriers to the wider adoption of social prescribing within the healthcare system.

### Lack of understanding and awareness

One challenge is the lack of understanding and awareness of available resources, both amongst healthcare professionals and patients. As social prescribing is a relatively new concept, healthcare providers may be unaware of the available services and may find it difficult to explain these to their patients (9). A survey on general practitioners' perceptions of social prescribing within the UK's National Health System (NHS) found that 63% of doctors felt that they would like a deeper understanding of the practice, with some responding that they were unaware of some aspects of social prescribing (27). In countries where general practitioners operate individually, they may feel overwhelmed by the lack of capacity to take on social prescribing amidst their already heavy workload and responsibilities (28).

### Patient expectation and acceptance

Many patients are unaware of the existence of social prescribing programs and their potential benefits (29, 30). In addition, patients who favor medical treatments may be less open to considering social prescribing programs (31). Many patients present to their doctors with preconceived expectations and personal agendas. For example, patients conventionally expect physicians to thoroughly assess their medical complaint, come to a diagnosis with appropriate investigations and formulate a treatment and long-term care plan (32). In fact, Williams et al. found that patients primarily wanted an explanation to their health problems and showed lesser desire for “support” (33). With this in mind, social prescribing should be advocated as an adjunctive measure for holistic care in the healthcare setting, and not as a replacement for medical therapy.

### Lack of evidence for social prescribing

Evidence-based medicine is the meticulous, explicit, judicious, and reasonable use of modern, best evidence in decision-making about the care of individual patients (34). It has grown in popularity due to its transparent nature and the extensive accessibility of electronic databases from which clinical data and research can be acquired (35). In this era where evidence-based medicine has helped to routinely define the standard of care in medical practice, the evidence base relating to social prescribing has been problematic and is a barrier to its widespread adoption (25).

Given that the practice of social prescribing is heterogenous and highly dependent on local context, it is difficult to evaluate its components and outcomes (25). It is also difficult to arrive at generalizable conclusions that can be applied to societies with vastly different cultures. A systematic review conducted in 2017 on the effectiveness of social prescribing programs found little evidence of either effectiveness or value for money (36). The relative lack of robust evidence on social prescribing may hinder its acceptance within the medical profession.

### Sustainability and funding

The administration of a robust social prescribing system requires many moving parts, including human resources and community programs. The goodwill of the voluntary sector is crucial to social prescribing as community services are often provided free-of-charge with volunteer help. In an evaluation of a social prescribing pilot in Brighton and Hove by Farenden et al., it was found that a total of 5824 volunteer hours were given to community navigation during the first 12 months of the pilot program (37). In addition, funding for social prescribing programs come from a combination of public and private funding, which are often non-recurrent and variable depending on governmental goals (38). As social prescribing becomes more popular, the operating costs to run and expand services to cope with more referrals can be expected to rise. In fact, the total expenditure on adult social care services by the NHS in England has risen over the past decade, with the total expenditure in 2022/23 more than £2 billion higher than in 2010/11 (39). Thus, its long-term sustainability remains uncertain and must be thoroughly evaluated.

## A paradigm shift in medical care

Social prescribing facilitates a shift in medical care towards a person-centric and preventive care approach. It acknowledges that a balanced lifestyle is as crucial as medical care and emphasizes the importance of proactively addressing SDOH in disease prevention. In the context of an aging and increasingly chronically unwell population, preventive interventions are key to ensuring a sustainable healthcare system (40).

## Conclusion

In conclusion, despite growing recognition of the potential benefits of social prescribing in family medicine, various challenges pose barriers to its widespread adoption. Thus, it is important to continue raising awareness of social prescribing and pursuing evaluation of its efficacy. By addressing the current barriers and promoting greater integration of social prescribing in family medicine, we can move towards embracing a more holistic approach to healthcare which embodies the “biopsychosocial” concept of medicine.
